# Case reports at the vanguard of 21^st^ century medicine

**DOI:** 10.1186/1752-1947-6-156

**Published:** 2012-06-14

**Authors:** Michael R Kidd, Deborah C Saltman

**Affiliations:** 1 Faculty of Health Sciences, Flinders University, Adelaide, Australia; 2 The University of Sydney, Sydney, Australia

## 

As doctors, working with our patients, we have the opportunity to make new discoveries every day about human existence, health and disease. Research in medicine often starts with observations made during patient encounters.

Each of us has an ethical responsibility to report our new discoveries and share our new knowledge with our peers. Well-written case reports will always be a source of inspiration for clinicians and scientists seeking new ideas about clinical care and research directions.

This is why in 2007, with a group of colleagues from around the world, we founded a new medical journal, the Journal of Medical Case Reports. We were surprised to discover that this was the world’s first international medical journal devoted to publishing case reports from all clinical disciplines. We decided to publish only those case reports that are the first of their kind to be published in the English language medical literature. Each published case must add something new to medical knowledge [1].

We also decided to encourage our authors to include patient perspectives where the patient describes their own experience of the disorder and their treatment. As Sir William Osler once wrote, “The best teaching of medicine is that taught by the patient.”

And we decided to publish open access, which means that the content of the journal is available free of charge through the Internet, to ensure that our case reports are easily and freely accessible to clinicians and researchers in every nation of the world [1].

In the five years since the launch of the journal we have published over 2,000 case reports. In 2011, case reports were downloaded from our journal’s web site over 1,500,000 times.

The rationale for the journal is easy. In this era of evidence-based practice, we need practice-based evidence. The basis of this evidence is the detailed information we obtain from each person that we see in our clinics; the information about individual people that informs both our daily clinical care and clinical research. Our aim is that every case report published in our journal will add valuable new information to the world’s medical knowledge.

So where do case reports fit into the new millennium? The end of the last century marked the pinnacle of our understanding of evidence and how it can be aggregated. Whilst current methods of aggregation allow for a cross-sectional view of medicine, they do little to enhance our knowledge of certain areas of clinical medicine. For example, those chronic and complex problems where cure is not the endpoint (such as the long term management of diabetes or hypertension), diagnosis and management of diseases which can significantly worsen within short intervals (such as many cancers), and the rise of personalised medicine and companion diagnostics (such as imantinib requiring a companion diagnostic test and then being prescribed to those who are genetically eligible).

The National Institutes of Health have recognised some of these hurdles in their work supporting clinical research. Challenges include the unmanageable number of interacting components within experimental and control interventions, the complexity of behaviours required by those clinicians delivering or receiving the intervention, the number and variability of clinical outcomes, and the degree of flexibility or tailoring of the intervention permitted in clinical settings.

Previously guidance and guidelines, chart audits and qualitative data collection assisted in filling in the some of the gaps. However they remain crude instruments in any armamentarium designed to describe “real-time” patients. Our inability to determine pathways of care still remain in the following areas:

· Interdependence of sequential events;

· Variability in treatment schedules, dosages and regimens;

· Concomitant or causal comorbidities;

· Contingency decision-making;

· Missing information from quantitative data, especially in complex sequencing of disease management, where it is difficult by other means to aggregate individual pathways, and where real life examples to assist clinicians with staging and treatment choices are few.

There is no doubt that case studies can assist us in meeting some of these challenges in the future. The current problem is not unlike that of evidence-based medicine in the previous century – how do we aggregate the data and/or describe it? It may be that consistency, stability and trends will become the new way of describing real life cases.

Case reports provide important and detailed information about an individual. This information can often be lost in research studies where individual results are aggregated. Case reports can also serve as an early warning signal of the adverse effects of new medications, or the presentations of new and emerging disease. And case reports can detail findings that can shed new light on the possible pathogenesis of a disease or an adverse effect [1]. Our authors are required to demonstrate what their case report adds to the medical literature. For example, is it the first report of its kind in the literature? Does it significantly advance understanding of a particular disease aetiology or drug mechanism? Does it have broad clinical impact across more than one clinical specialty?

Yet case reports have become a neglected area for publication. This is at least partly due to the impact of Impact Factors. Some case reports may not receive high numbers of citations and this drives many journals to decide not to publish case reports. Yet some of the most highly cited publications in the history of medical publishing have involved case reports and case series, for example the first ever report of people with AIDS in the United States of America published in 1981 in the Morbidity and Mortality Weekly Report of the Centres for Disease Control in the United States of America [2].

Since the foundation and launch of Journal of Medical Case Reports in 2006, its aims, scope and principles have been adopted by other publishers who have launched other new journals. Most notably, the BMJ Group launched BMJ Case Reports, and the field has grown with the International Journal of Surgery Case Reports (Elsevier), the Journal of Surgical Case Reports (JSCR Publishing), and the International Medical Case Reports Journal (Dove Press). We are proud and pleased to be spearheading such a significant change in medical publishing.

We believe that it is time for case reports to be considered first class citizens in the medical literature. We are committed to ensuring the quality of our publication through the quality and likely clinical impact of the case reports and case series published in the Journal of Medical Case Reports, high standards of open peer review and indexing with PubMed.

The case reports we publish have the potential to contribute to research and change clinical practice. Accurate recounting of clinical experience continues to be essential to the progress of medicine. For example we have received a number of case reports related to patients who presented with new or re-emerging diseases. Recently we published a series of case reports from the 2009 H1N1 influenza pandemic [3-6].

Case reports can also be used to report medical errors. The lessons obtained from medical errors can be important in preventing similar problems for future patients. As an example our journal recently published the first case report in the medical literature of acute renal failure secondary to the accidental administration of a high dose of indomethacin to a neonate [7].

We have been pleased to receive many case reports from recent medical graduates. Case reports provide an opportunity for medical students and recent graduates to start conducting research by writing up their own clinical observations about individual patients as part of their training in evidence-based practice.

We thank our deputy editors and editorial board members, our many authors and peer reviewers and the editorial staff at BioMed Central. We especially thank the many patients and their family members who have provided consent to have details of medical histories and clinical care published in our journal. And our special thanks to those patients who have shared their own insights through submitting written perspectives about their conditions.

To mark our five year milestone, we invite our readers to assist us in working with our published case reports to aggregate the now amassing information that is contained within them. Publications in this area will be welcomed.

We hope that the case reports in our journal, and the data they contain, will continue to assist our colleagues in their daily clinical work and also serve as a source of inspiration for clinical researchers seeking ideas about new research directions. It is a motivation for us all to know that what we observe and report today may contribute to the health and well being of many other people in the future.
